# A quality improvement project focused on improving the completion of ‘notification of diagnosis’ forms for the dementia register, in an outpatient setting

**DOI:** 10.1192/bjo.2021.141

**Published:** 2021-06-18

**Authors:** Katherine Johnston, Lauren Megahey

**Affiliations:** Southern Health and Social Care Trust

## Abstract

**Aims:**

The dementia register is designed to keep a record of all patients diagnosed with mild cognitive impairment (MCI) or dementia. Following diagnosis, a ‘notification of diagnosis’ form should be completed and the patient added to the register. The register is used to collate figures and to assess capacity and demand on services.

Our baseline audit revealed suboptimal completion of these forms, therefore we initiated a quality improvement project. Our aim was to achieve completion of the ‘notification of diagnosis’ forms in 50% of new memory patients seen in clinic and diagnosed with MCI or dementia, within 3 months.

**Method:**

A baseline audit of a random sample of 52 patients, from the 380 patients on the memory clinic list was analysed. 40 of these 52 patients had a diagnosis of MCI or dementia and when cross-matched with the dementia register, only 12 (30%) of the 40 were on the register.

We designed an improvement project which focused on improving awareness of the process and facilitating ease of completion of the form, for example, by placing the form in all new patients’ notes. Our results were then monitored and reviewed on a monthly basis for 3 months, to assess the impact of these changes.

**Result:**

Each month, the percentage completion of the 'notification of diagnosis' forms was calculated for new memory patients diagnosed with MCI or dementia in clinic. In the first month of the project (November 2020), 75% completion of forms was achieved. This was a significant improvement from baseline. In December, 66.7% completion was achieved (plus one patient was already on the register) and in January 2021, there was 50% completion (plus one patient was already on the register).

**Conclusion:**

The results showed an improvement in the completion of forms from baseline, and we did reach the initial aim set at 50% by 3 months. However, the trend of the results showed a steady decline in percentage completion of forms over the 3 month period. We noted that over time the forms were no longer consistently placed in the new patients’ notes, reducing accessibility to the forms. Other potential factors include a reduction in enthusiasm/ a decline in prioritisation of this project over time. Suggestions have been made to facilitate ongoing improvement and the results will continue to be reviewed.

